# Potential distribution of three invasive agricultural pests in China under climate change

**DOI:** 10.1038/s41598-024-63553-3

**Published:** 2024-06-13

**Authors:** Yanjing Zhang, Yaqiong Wan, Chenbin Wang, Jing Chen, Qin Si, Fangzhou Ma

**Affiliations:** 1 State Key Laboratory of Environmental Protection and Biosafety, Nanjing Institute of Environmental Sciences, Ministry of Environmental Environment, 8 Jiangwangmiao Road, Nanjing, 210042 China; 2 Jiangsu Maritime Institute, 309 Gezhi Road, Nanjing, 211100 Jiangsu China

**Keywords:** Invasive agricultural insects, MaxEnt, Climate change, Environmental variables, Habitat distribution, Ecology, Zoology

## Abstract

Invasive pests reduce biodiversity and ecosystem service functions, thereby leading to economic and also agricultural losses. Banana skipper (*Erionota torus Evans)*, red palm weevil (*Rhynchophorus ferrugineus*), and coconut caterpillar (*Opisina arenosella* Walker) are invasive insect pests in the palm-growing regions and they have had serious consequences for the planting of bananas (*Musa nana*), palms (*Trachycarpus fortune*) and coconut (*Cocos nucifera*). Based on screened occurrence data, the present research utilized Maximum Entropy model (Maxent) to simulate the distribution dynamics of these three invasive insects in China, under current and future climate (2050s, 2070s, 2090s) in two shared socio-economic pathways (SSPs: 126 and 585) of the newly released coupled model intercomparison project phase6 (CMIP6). The results show that: (1) Under current and future climate conditions, all model groups exhibited an AUC value exceeding 0.92, which shows that the model prediction results are very good;(2) The suitable habitat area of *E. torus Evans* remains relatively stable with some expansion in the SSP126 of 2090s and some contraction in the SSP585 of 2090s. The suitable habitat area of *R. ferrugineus* showed an overall contraction, with substantial contraction in the SSP585 of 2090s.The suitable habitat area of *O. arenosella* has an overall expansion, with the most pronounced expansion in the SSP585 of 2070s; (3) The current centroid of suitable habitats for *R. ferrugineus* and *E. torus Evans* is located in Guangxi Province and wholely shift toward the south direction under future climate. The centroid of suitable habitats for *O. arenosella* is currently located in the northeastern maritime area of Hainan Province and will shift toward the north direction under future climate; (4) Temperature, precipitation and Human disturbance factors (Population density and Human influence index) were crucial variables for describing the distribution of the three species. For *E. torus Evans* in particular, percentage contributions of Population density was up to 31.4, which is only 0.1 different from ranked first Bio19 (Precipitation of the coldest quarter). The dynamics of habitats of these three species and the correlating driver factors proposed in this work provide essential insights into future spatial management of the three invasive insects in China. Our work is necessary and timely in identifying newly areas at high risk of expansion of the three invasive insects in the future, then suggesting strategic control measures to prevent their spread, and finally providing scientific evidence for the early prevention and rapid response to the three invasive insects.

## Introduction

Invasive alien species are a global environmental concern^1,2^. They pose a significant threat to native species and have potentially severe ecological and socioeconomic consequences^3–6^. As global economic integration accelerates, trade and transportation sectors are expanding, leading to more frequent alien invasions and the damage they create^7^. Invasive pests also reduce biodiversity and ecosystem service functions, leading to economic and environmental losses, extinction of species and threats to human health^8^. Invasive pests of agriculture have significant negative impacts on crops due to their competitiveness and environmental adaptability. The import and export of agricultural products facilitates the invasion of agricultural pests^9^. Invasive agricultural pests have caused serious damage to key crops including bananas (*Musa nana*), palms(*Trachycarpus fortune*)and coconut (*Cocos nucifera*)in China.

*Erionota torus Evans* (Lepidoptera: Hesperiidae), is a common banana pest. Its distribution was previously confined to mainland Southeast Asia until 1971, when it was identified in Okinawa^10^. In 1986 it was found infesting banana foliage on the experimental grounds of Taiwan^10^. Its distribution has since expanded and now includes Southeast Asia and Pacific regions, ranging from Papua New Guinea and Australia, Sikkim to south China, Myanmar, Malaysia, and Vietnam. It is extensively distributed in the southern and southeastern coastal regions of China^11–14^. Virtually all banana varieties are vulnerable to damage caused by this skipper^15^. The larvae roll and feed on banana leaves, causing substantial damage^10,16^. This pest poses a challenge to the banana industry of China and reduces annual banana production by an estimated 10–30%^17^. *Rhynchophorus ferrugineus* (Coleoptera: Curculionidae) is a devastating pest to palm trees^18–20^. *R. ferrugineus*, which originated in southern Asia and Melanesia, now has a broad distribution spanning Oceania, Asia, Africa, and the Americas^21^. In China, it was first noted in Zhongshan City, Guangdong Province, in 1997, and it has since invaded southern provinces such as Hainan, Guangxi, and Yunnan. It is now widely distributed across extensive areas in the southern region of China^22,23^. Adult females deposit eggs in wounds or openings located at the palm crown or leaf scars^24^. The larvae feed on the soft palm tissue. Their sustained feeding activity produces tunnels along the stem. The pupae inside oval cocoons made from palm fibers^25^. The large area of palm plants planted in southern coastal provinces of China is heavily threatened by *R. ferrugineus*, which has had a negative impact on the agroforestry ecosystem in these areas^26–28^. Outbreaks of *R. ferrugineus* in China have severely impacted the coconut and areca nut industries, and this species threatens the ecological stability of the coastal area of China^29^. *Opisina arenosella* Walker (Lepidoptera: Xyloryctidae) is another significant pest of coconut palms. It was initially identified in India and Sri Lanka in the mid-nineteenth century. *O. arenosella* has since spread to Myanmar, Bangladesh, Thailand, Malaysia, Vietnam, and Pakistan^30,31^. In 2013, it was found in Wanning City (Hainan) and has spread to Guangdong, Guangxi, and Fujian^32^. The larvae feed on the epidermis and mesophyll of coconut leaves and construct a tunnel underneath the leaf using their feces and silk. On leaves that have been consumed by the larvae, only the upper epidermis remains, giving it a burnt appearance^33,34^. Seriously, the entire crown of the coconut tree can be damaged in a short period of time, resulting in the death of the tree body and endangering the safety of agricultural production^35,36^. The damage inflicted by larvae reduces plant yield and results in significant economic losses^36,37^. Owing to the above three invasive insects produced damaging agroforestry and causing economic losses, there is an urgent need to study the potential geographical distribution of them in China for implement proactive measures for early monitoring and prevention and control.

The spread of insects is affected by temperature, rainfall, wind currents, etc. and the effects of global climate change are particularly pronounced^38^. Global warming affects insect distribution patterns, growth, and development, phenological synchronization with host plants, egg-laying rates, and genetic composition^39^. The National Center for Atmospheric Research indicates that increased greenhouse gas emissions and ozone layer depletion are contributing to an increase in the surface temperature of the Earth^9,40^, with China experiencing a temperature rise between 1.6 to 5.0 °C^41^. Warming climate implies expand the distribution of favorable habitats into higher latitudes^38,42^, but this is not the case for all invasive species. There are studies that have found global warming might decrease the potential global distribution area of *Solenopsis. geminata*^43^. In addition to climatic factors, environmental variables such as Population density, Human influence index, Land cover dataset, Normalized difference vegetation index, and Land use and cover change are also important factors affecting the distribution of invasive species^43–45^ Human activities have especially a great influence on insect distributions^44^. Global trade and transportation are primary pathways for the spread of invasive insects. Only by strengthening the inspection and quarantine of invasive species can we reduce the spread of invasive insects^44,45^. There are also numerous terrain factors to consider when predicting the distribution of invasive insects^10,44,48,49^. These include altitude, slope, and slope direction. Predicting the potential distribution of invasive species under climate change scenarios holds practical importance, both for monitoring of areas subject to invasion and for early detection in areas that have not yet been affected^45^.

Ecological niche models (ENMs) predict environmental suitability and distribution of species by integrating species distribution data with environmental variables, such as climate and the environment^46^. The ENMs include GARP, CLIMEX, Biomod2, and MaxEnt^47^. Recognized for its precision, operational stability, and efficiency, MaxEnt based on the maximum entropy model have high predictive capacity than other models, being particularly resilient to sample bias^42^. MaxEnt has been widely used to predict suitable areas of invasive pests, such as *S. geminata*^43^, S*olenopsis invicta*^46,48^, *Wasmannia auropunctata*^49^, *Bemisia tabaci*^50^. Most studies are based on the Coupled Model Intercomparison Project Phase 5 (CMIP5), while the new version, CMIP6, has higher climate sensitivity and allows a broader exploration of future outcomes^51^. Therefore, CMIP6 affords improved consideration of the environmental factors influencing species distribution and provides a more accurate distribution prediction for invasive alien insects.

Given few studies have investigated areas that might become suitable for *E. torus Evans*, *R. ferrugineus*^22^, *and O. arenosella* under different global warming scenarios by MaxEnt and in order to fill the gap in the existing literature, this study used the Maxent model to predict the potential distribution and centroid shifts of *E. torus Evans*, *R. ferrugineus*, *and O. arenosella* under current and future (2050s, 2070s, 2090s) climate scenarios (SSP126, SSP585) in China. The primary environmental variables influencing these invasions trajectories were examined. This study aimed to provide guidance for the formulation of policies on invasive pest control and establish a foundation for improved management of these invasive species.

## Materials and methods

### Occurrence data of the three species

**Figure 1:**
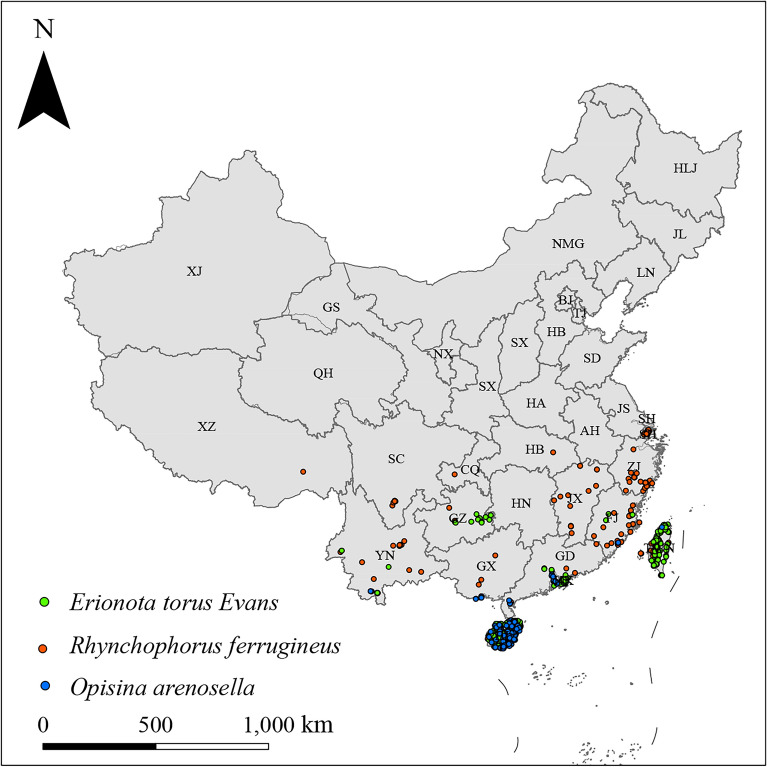
Occurrence records of *Erionota torus Evans, Rhynchophorus ferrugineus* and *Opisina arenosella* walker in China. Note: AH (Anhui); BJ (Beijing); CQ (Chongqing); FJ (Fujian); GD (Guangdong); GS (Gansu); GX (Guangxi); GZ (Guizhou); HA (Henan); HB (Hubei); HE (Hebei); HI (Hainan); HK (Hong Kong); HL (Heilongjiang); HN (Hunan); IM (Inner Mongolia); JL (Jilin); JS (Jiangsu); JX (Jiangxi); LN (Liaoning); MO (Macao); NX (Ningxia); QH (Qinghai); SC (Sichuan); SD (Shandong); SH (Shanghai); SN (Shaanxi); SX (Shanxi); TJ (Tianjin); TW (Taiwan); XJ (Xinjiang); XZ (Tibet); YN (Yunnan); ZJ (Zhejiang).

A total of 513 occurrence records of the three species in China (172, 229, and 112 for *E. torus* Evans, *R. ferrugineus* (Oliver, 1790), and *O. arenosella* Walker, respectively) were obtained from various sources (Fig. 1). The sources included: (1) the Global Biodiversity Information Facility (GBIF) website (http://www.gbif.org/); (2) published studies; and (3) species distribution reports from authoritative news outlets. To ensure the accuracy of species occurrence records and mitigate the impact of sampling bias, three-step screening of the collected occurrence records was performed using ArcGIS. Duplicate occurrence records within a raster with a 2.5-min resolution were deleted. Occurrence records that were not distributed on land were removed. Lastly, incorrect occurrence records were discarded. The spatial resolution of the environmental variables used in this study was 2.5 arc-minutes, and the coverage area was approximately 21.0 km^2^; thus the buffer radius was set to 2.5 km for consistent with the resolution of the environment variable^52^. This step of screening can reduce the sampling bias and improve the prediction accuracy of the model.

### Environmental data

Nineteen grid-based bioclimatic variables used in this study were downloaded from a WorldClim (www.worldclim.org) dataset. The current climate variables used were based on historical data from 1970 to 2000, updated to WorldClim 2.1 in March 2020. WorldClim 1.4 was based on historical data from 1960 to 1990. SSPs (Shared Socio-economic Pathways) were calibrated with WorldClim 2.1 as the baseline and proposed in IPCC-CMIP6. The model predicted by WorldClim2.1 might have better stability than WorldClim 1.4^51^. Future climate data were designated for three time periods: 2050s, 2070s, and 2090s. Each time period is 20 years apart. Two shared socioeconomic pathways (SSP126 for the moderate scenario and SSP585 for the pessimistic scenario) of the Sixth Phase of the Coupled Model Intercomparison Project (CMIP6) under the Beijing Climate Center Climate System Model (BCC-CSM2-MR) were selected for use in modeled projections. SSP126 means that a world of sustainability-focused growth and equality, radiative forcing stabilizes at 2.6 W/m^2^ in 2100. SSP585 means a world of rapid and unconstrained growth in economic output and energy use, radiative forcing stabilizes at 8.5 W/m^2^ in 2100^51^.

Insects are sensitive to the environmental changes, those induced by topographic factors and human disturbances^53–56^. Therefore, in addition to the 19 climate variables mentioned above, we incorporated terrain factors, human disturbance factors, and habitat factors in the analysis. The Population density (PD) and the Human Impact Index (HII) were obtained from socioeconomic data and application centers (SDAC, http://sedac.ciesin.columbia.edu). The China Land cover data (LC) were obtained from the ESA Data User Element (http://due.esrin.esa.int/page_globcover.php). The Land use and cover change (LUCC) and Normalized difference vegetation index (NDVI) were downloaded from the Resource Environmental Science and Data Center of the Institute of Geographic Sciences and Natural Resources Research, Chinese Academy of Sciences (https://www.resdc.cn/). The digital elevation model (DEM) data were downloaded from the Geospatial Data Cloud (https://www.gscloud.cn/). The slope and aspect data were extracted from the DEM. A total of 27 environmental variables were selected for this study, including 19 bioclimatic variables, PD, CLC, HII, LUCC, NDVI, DEM, slope, and aspect (Table 1).

**Table 1:** Environmental variables related to the distribution of *E. torus Evans*, *R. ferrugineus*, *and O. arenosella.*

Factor	Code	Description	Unit	Weather to use *Rhynchophorus ferrugineus for Modeling*	Weather to use *Erionota torus Evans for Modeling*	Weather to use *Opisina arenosella* walker *for Modeling*
Climate factor	bio1	Annual mean temperature	℃	No	Yes	No
	bio2	Mean diurnal range	℃	Yes	Yes	Yes
	bio3	Isothermality	℃	No	Yes	No
	bio4	Temperature seasonality	/	Yes	No	No
	bio5	Maximum temperature of the warmest month	/	No	No	No
	bio6	Minimum temperature of the warmest month	℃	No	No	Yes
	bio7	Annual mean temperature range	℃	No	Yes	No
	bio8	Mean temperature of the wettest quarter	℃	Yes	No	Yes
	bio9	Mean temperature of the driest quarter	℃	Yes	No	No
	bio10	Mean temperature of the warmest quarter	℃	Yes	No	No
	bio11	Mean temperature of the coldest quarter	℃	No	No	No
	bio12	precipitation	mm	No	Yes	No
	bio13	Precipitation of the wettest month	mm	No	Yes	No
	bio14	Precipitation of the driest month	mm	Yes	No	No
	bio15	Precipitation seasonality (CV)	/	Yes	No	Yes
	bio16	Precipitation of the wettest quarter	mm	Yes	No	No
	bio17	Precipitation of the driest quarter	mm	No	No	No
	bio18	Precipitation of the warmest quarter	mm	No	No	Yes
	bio19	Precipitation of the coldest quarter	mm	No	Yes	Yes
Terrain factor	DEM	digital elevation model data	m	Yes	Yes	Yes
	Aspect	Aspect	°	No	No	Yes
	Slope	Slope	°	Yes	Yes	No
Disturbance factor	PD	Population density	inhabitants/km^2^	Yes	Yes	Yes
	HII	Human influence index	/	Yes	Yes	Yes
Habitat factor	LUCC	Land use and cover change	/	Yes	Yes	Yes
	NDVI	Normalized difference vegetation index	/	Yes	Yes	Yes
	LC	China Land cover data	km^2^	Yes	Yes	Yes

To facilitate the spatial analysis within China, all variables listed above were resampled to 2.5 arc-min (approximately 4.5 km^2^) by using the spatial analysis tools in ArcGIS 10.6. Generally, the higher the spatial resolution of the environmental layer is, the more accurate the model running result is. However, the pressure of the model running will greatly increase^51^. Therefore, we chose the 2.5 min spatial resolution of all environmental layers to ensure high accuracy and good running ability. Excessive environmental variables can amplify the dimensions of ecological space and hinder model predictions. Thus, a meticulous selection of environmental variables was executed in this study. Initially, the “Extract Multi Values to Points” tool in ArcGIS was used to extract 27 environmental factors at the sample points. Subsequently, the “ENMTools” package in R was utilized for conducting correlation analysis. We filtered for environment variables by integrating Jackknife importance values and removing environmental variables with a correlation coefficient |r| ≥ 0.80 and negligible contributions to the model^57^.

### ENM model

The invasive species occurrence records and environmental factors were imported into the MaxEnt model (Maxent version 3.4.4) to predict the current and future suitable areas for each species as illustrated. The commonly used model evaluation indexes include Cohen’s kappa (KAPPA) values, the true skill statistic (TSS), and area under the receiver operating characteristic (ROC) curve (AUC). Kappa is affected by species distribution rate^58^. TSS equally weights sensitivity and specificity, but different weights may be required for practical application^59^. The AUC values not affected by thresholds are more objective than others for model assessment. Hence, the MaxEnt model predictions are assessed for accuracy using the AUC values. The AUC criteria are defined as follows: poor (AUC < 0.75), good (AUC 0.85–0.95), and excellent (AUC > 0.95)^60^. In MaxEnt, 25% of the data are randomly allocated as a test set (with 75% as the training set), and model robustness is verified through 15 repetitions. The maximum number of iterations is set at 5000, and the background points are set to 10,000 to seek the optimal solution, while other parameters remain at default values.

### Evaluation of the dynamics of species habitat suitability

Upon completing the modeling process, we generated habitat suitability maps for both the present and anticipated future scenarios using Maxent’s outputs. The categorization of habitat suitability was based on the maximum training sensitivity plus specificity logistic threshold (MTSS): Unsuitable habitat (< MTSS): Regions not favorable for species establishment. Minimum suitable habitat (MTSS-0.4): Basic survival conditions met. Moderately suitable habitat (0.4–0.6): Conducive for moderate growth. Highly suitable habitat (> 0.6): Optimal conditions for thriving populations. We selected the average “MTSS” to identify the threshold value, because this selection criteria had the highest specificity and sensitivity and could produce more accurate predictions than widely used 0.5 as fixed threshold and kappa-maximizer criteria^61^. A higher value for suitability indicates a greater likelihood of the potential distribution of the predicted species at the site. To understand potential shifts in habitat suitability, we compared the current distribution with projections for 2050s, 2070s, 2090s.

To provide a more intuitive representation of potential changes in the habitat suitability of invasive insects in China under different climate scenarios, we calculated the proportion and area of different suitability zones using the grid layer attribute table in ArcGIS software. The SDM tool was used to analyze the stability (Compared to the present, the area is also suitability zones in the future), expansion (Compared to the present, the area is expanded suitability zones in the future), contraction areas (Compared to the present, the area is shrinking suitability zones in the future), and the centroid shift of the invasive insect habitat suitability zones.

Centroids play a key role in describing the spatial distribution of terrestrial classes. The accumulation, dispersion, and migration of terrestrial types can be reflected by centroid shifting in different periods^62^. The raster maps of suitable habitats of invasive species were converted to vector maps, and each centroid position and transfer distance of suitable habitats in different periods were calculated using the ArcGIS classification statistical tool^63^. By calculating the center of mass for the distribution of each species, we inferred potential distribution shifts and derived insights into the direction and magnitude of these changes^64^.

## Results

### Screened occurrences and environment variables

In order to avoid overfitting, we performed a series of screenings on the distribution points of three invasive insects. Consequently, 97, 173, and 72 occurrence points for *E. torus Evans*, *R. ferrugineus*, and *O. arenosella*, respectively, were retained for model predictions. A set of optimized environmental variables was finalized for constructing predictive models for each of the three species (Table 1).

### Current potential distribution

**Figure 2:**
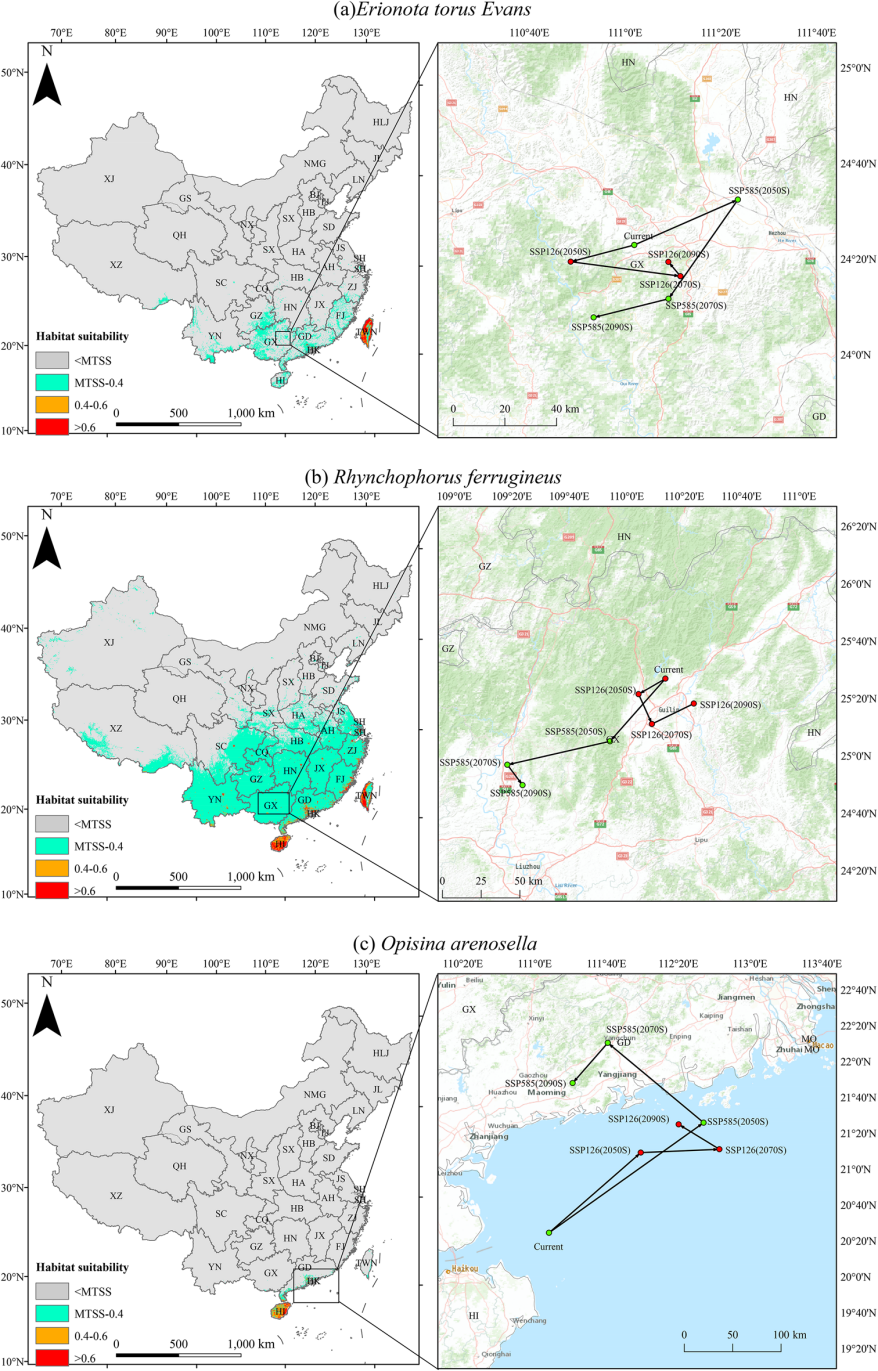
The current potential distribution (Left) and the centroid distribution transfer (Right) in three future periods (2050s, 2070s, 2090s) under SSP126 and SSP585 scenarios for (**a**) *E. torus Evans*, (**b**) *R. ferrugineus*, (**c**) *O. arenosella*. Arrow indicates magnitude and direction of predicted change with the change of time (2050s, 2070s, 2090s).

The current total suitable habitat area of *E. torus Evans* is approximately accounting for 2.99% of the total land area. Of this, highly suitable habitat is roughly 0.18% of the total land area. These highly suitable habitats are predominantly found in Taiwan, with sporadic distributions in select regions of Guangxi, Guangdong, and Fujian. The moderately suitable habitats, covering 0.11%, are distributed near and around highly suitable habitats (Fig. 2a, Table 2).

**Table 2:** Suitable areas change for *E. toru Evans*, *R. ferrugineus* and *O. arenosella* under different climate change scenarios.

Species	*Erionota toru Evans*							*Rhynchophorus ferrugineus*							*Opisina arenosella* walker						
Scenario		SSP126			SSP585				SSP126			SSP585				SSP126			SSP585		
Suitable habitats																					
Time period	Current	2050s	2070s	2090s	2050s	2070s	2090s	Current	2050s	2070s	2090s	2050s	2070s	2090s	Current	2050s	2070s	2090s	2050s	2070s	2090s
High-suitable habitats																					
Area/km^2^	1.73	1.55	1.83	1.77	1.86	2.38	2.53	4.25	4.96	2.63	3.91	1.99	1.93	1.15	0.73	2.12	1.18	2.39	2.24	4.67	1.15
Area changing/km^2^		− 0.18	0.1	0.04	0.13	0.65	0.8		0.71	− 1.62	− 0.34	− 2.26	− 2.32	− 3.1		1.39	0.45	1.66	1.51	3.94	0.42
Percentage	0.18%	0.16%	0.19%	0.18%	0.19%	0.25%	0.26%	0.44%	0.52%	0.27%	0.41%	0.21%	0.20%	0.12%	0.08%	0.22%	0.12%	0.25%	0.23%	0.49%	0.12%
Middle-suitable habitats																					
Area/km^2^	1.07	1.23	1.23	1.17	1.17	1.16	1.32	5.45	5.95	5.38	6.73	5.19	5.7	3.53	2.00	2.01	2.27	2.42	2.64	3.81	3.81
Area changing/km^2^		0.16	0.16	0.1	0.1	0.09	0.25		0.5	− 0.07	1.28	-0.26	0.25	− 1.92		0.01	0.27	0.42	0.64	1.81	1.81
Percentage	0.11%	0.13%	0.13%	0.12%	0.12%	0.12%	0.14%	0.57%	0.62%	0.56%	0.70%	0.54%	0.59%	0.37%	0.21%	0.21%	0.28%	0.25%	0.27%	0.40%	0.40%
Low-suitable habitats																					
Area/km^2^	25.87	24.84	25.72	35.07	25.79	23.02	19.44	215.95	77.73	63.49	84.62	70.44	63.39	53.03	1.91	2.91	2.92	3.63	3.53	8.27	7.41
Area changing/km^2^		− 1.03	− 0.15	9.20	− 0.08	− 2.85	-6.43		− 138.22	− 152.46	− 131.33	− 145.51	− 152.56	− 162.92		1.00	1.01	1.72	1.62	6.36	5.50
Percentage	2.69%	2.59%	2.68%	3.65%	2.69%	2.40%	2.03%	22.50%	8.10%	6.61%	8.81%	7.34%	6.60%	5.52%	0.20%	0.30%	0.30%	0.38%	0.37%	0.86%	0.77%
Non-suitable habitats																					
Area/km^2^	931.33	932.38	931.22	921.98	931.18	933.43	936.71	734.35	871.36	888.51	864.74	882.37	888.98	902.29	955.36	952.78	953.18	951.55	951.59	943.25	947.63
Area changing/km^2^		1.05	− 0.11	− 9.35	− 0.15	2.10	5.38		137.01	154.16	130.39	148.02	154.63	167.94		− 2.580	− 2.180	− 3.810	− 3.770	− 12.110	− 7.730
Percentage	97.01%	97.12%	97.00%	96.04%	97.00%	97.23%	97.57%	76.50%	90.77%	92.55%	90.08%	91.91%	92.60%	93.99%	99.52%	90.25%	92.29%	99.12%	99.12%	92.60%	93.99%
Total-suitable habitats																					
Area/km^2^	28.67	27.62	28.78	38.02	28.82	26.57	23.29	225.65	88.64	71.49	95.26	77.63	71.02	57.71	4.64	7.04	6.82	8.45	8.41	16.75	12.37
Area changing/km^2^		− 1.05	0.11	9.35	0.15	− 2.1	− 5.38		− 137.01	− 154.16	− 130.39	− 148.02	− 154.63	− 167.94		2.4	2.18	3.81	3.77	12.11	7.73
Percentage	2.99%	2.88%	3%	3.96%	3%	2.77%	2.43%	23.50%	9.23%	7.45%	9.92%	8.09%	7.40%	6.01%	0.48%	0.73%	0.71%	0.88%	0.88%	1.75%	1.29%

"Area" indicates the area of the corresponding suitable area under current and future climate; "area changing" indicates the area changed compared with the current climate conditions; "Percentage" refers to the proportion of various suitable habitats to the total area under current and future climate; SSPs: Shared socioeconomic pathways. Future changes in suitable habitats.

The current total suitable habitat area of *R. ferrugineus* is estimated to making up approximately 23.50% of the total land area. The highly suitable habitats are accounting for 0.44% of the total land area. These habitats are primarily located in central and southern Yunnan, Hainan Island, Taiwan, and the southeastern coastal regions. The moderately suitable habitats, making up 0.57%, are found surrounding the highly suitable habitats (Fig. 2b, Table 2).

The current total suitable habitat area for the *O. arenosella* is approximately 0.48% of the total land area. The highly suitable habitats are accounting for around 0.08% of the total land area. These habitats are mainly located in most parts of Hainan Island, with sporadic areas along the coastal regions of Guangxi and Guangdong. The moderately suitable habitats, covering 0.21%, are distributed similarly to the highly suitable habitats and are primarily on Hainan Island (Fig. 2c, Table 2).

**Figure 3:**
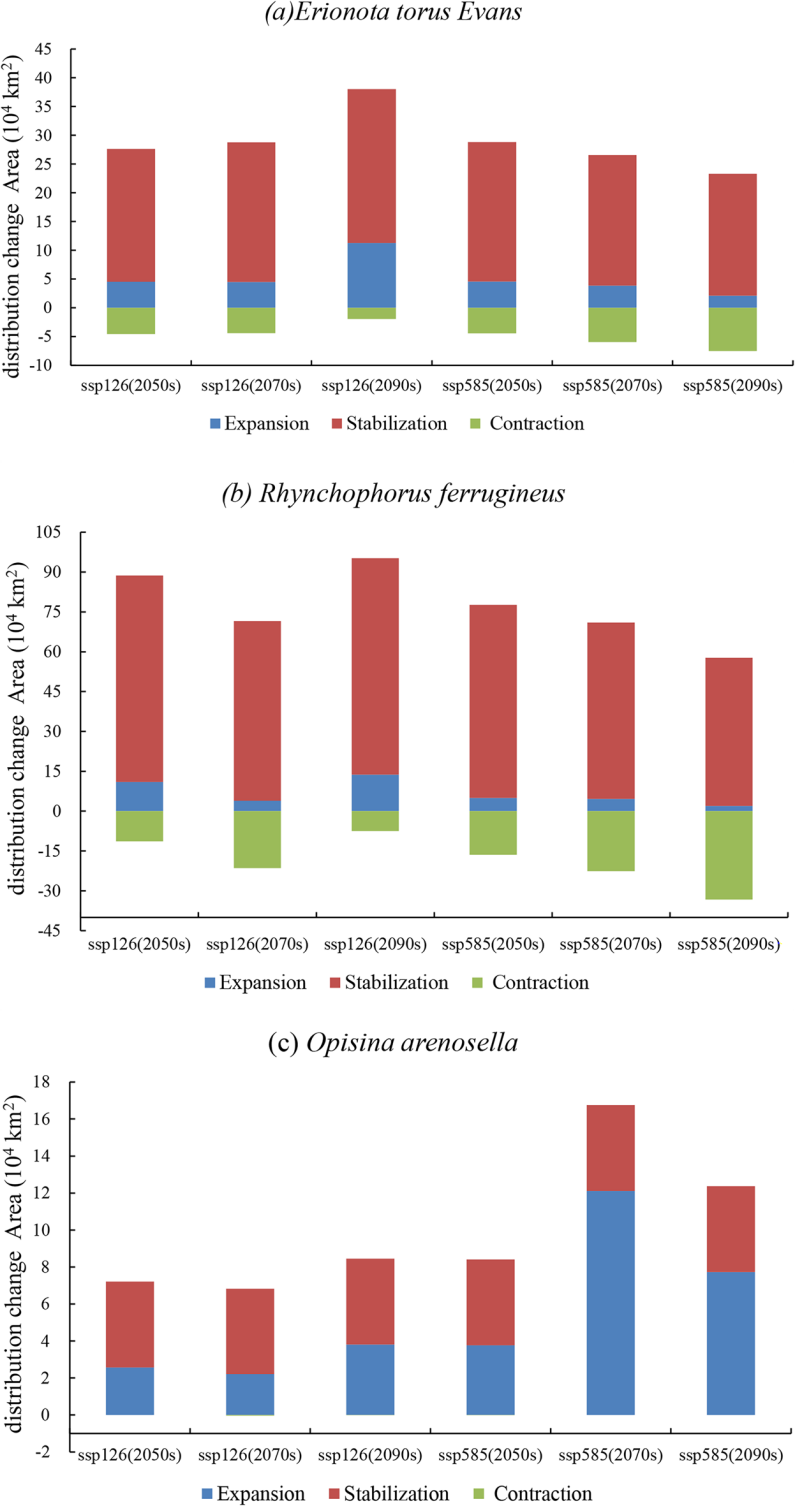
The projected distribution habitat area in three periods (2050s, 2070s, 2090s) showing expansion, contraction or stabilization under SSP126 and SSP585.

Under the future climate scenarios of SSP 126 and SSP 585 (2050s, 2070s, 2090s), there are varying degrees of changes in the suitable habitat areas of the three invasive insects (Table 2, Fig. 3). The suitable habitat of *E. torus Evans* exhibited overall stability with some significant expansion in the SSP126 (2090s) scenario and substantial contraction in the SSP585 (2090s) scenario. The majority of the suitable habitat for *R. ferrugineus* showed overall contraction, with substantial contraction in the SSP585 (2090s) scenario. The suitable habitat of *O. arenosella* demonstrated a notable overall expansion trend, with the most pronounced expansion observed in the SSP585 (2070s) scenario.

**Figure 4:**
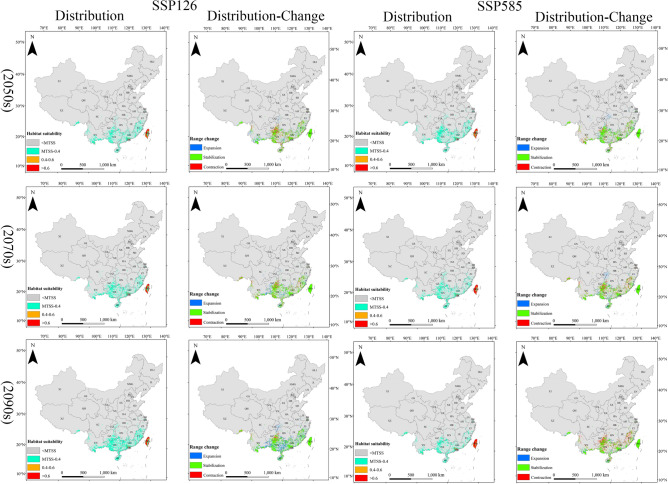
Projected future distributions of periods (2050s, 2070s, 2090s) for *E. torus Evans* and the changes in areas between future and current distributions under two scenarios (SSP126 and SSP585).

In the context of climate change, the potential suitable habitats for the *E. torus Evans* in China are primarily concentrated in Hainan, Guangdong, Guangxi, Yunnan, southeastern Guizhou, southeastern Tibet, Fujian, and Jiangsu (Fig. 4). In the SSP126 scenario, the potential suitable habitat area of the *E. torus Evans* slightly decreased in 2050s, followed by an increase in 2070s and 2090s, resulting in an overall net increase primarily due to the expansion of low-suitability regions. In the SSP126 (2090s) scenario, the maximum increase in low-suitability regions was observed, with an expansion of 9.20 km^2^. However, in the SSP585 scenario, the potential suitable habitat area slightly increased in 2050s but decreased in 2070s and 2090s, leading to an overall net decrease mainly due to the reduction in low-suitability regions. In the SSP585 (2090s) scenario, the most significant reduction was observed in the low-suitability regions, amounting to 6.43 km^2^. Overall, the total area of suitable habitat for *E. torus Evans* remained relatively stable, with slight changes occurring in the peripheral regions (Table 2).

**Figure 5:**
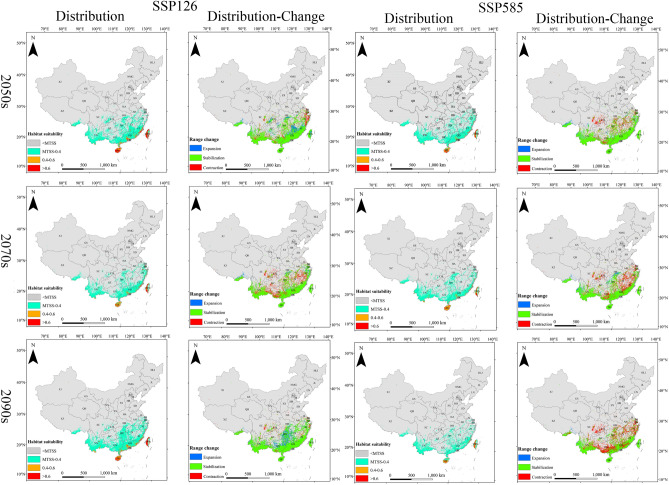
Projected future distributions of periods (2050s, 2070s, 2090s) for *R. ferrugineus* and the changes in areas between future and current distributions under two scenarios (SSP126 and SSP585).

Under the future climate scenarios, the potential suitable habitats for *R. ferrugineus* in China are mainly found in Hainan, Taiwan, Guangdong, Guangxi, Yunnan, Guizhou, southeastern Tibet, Sichuan, Chongqing, Hubei, Jiangsu, Anhui, Fujian, Jiangxi, and Zhejiang (Fig. 5). There is an overall decrease in the potential suitable habitat area for *R. ferrugineus* primarily due to a significant reduction in low-suitability areas and a notable increase in non-suitable areas. Under the SSP 126 (2070s) scenario, the most significant reduction in the low-suitability area was − 152.46 km^2^, while the most significant increase in non-suitable area was 154.16 km^2^. Similarly, under the SSP585 scenario, there was an overall decrease in the potential suitable habitat area, characterized by a significant reduction in low-suitability areas and a notable increase in non-suitable areas. The most significant reduction in the low-suitability area under the SSP585 (2090s) scenario was − 162.92 km^2^, while the most significant increase in the non-suitable area was 167.94 km^2^. Overall, the total suitable habitat area for *R. ferrugineus* is decreasing, with a gradual reduction at the borders (Table 2).

**Figure 6:**
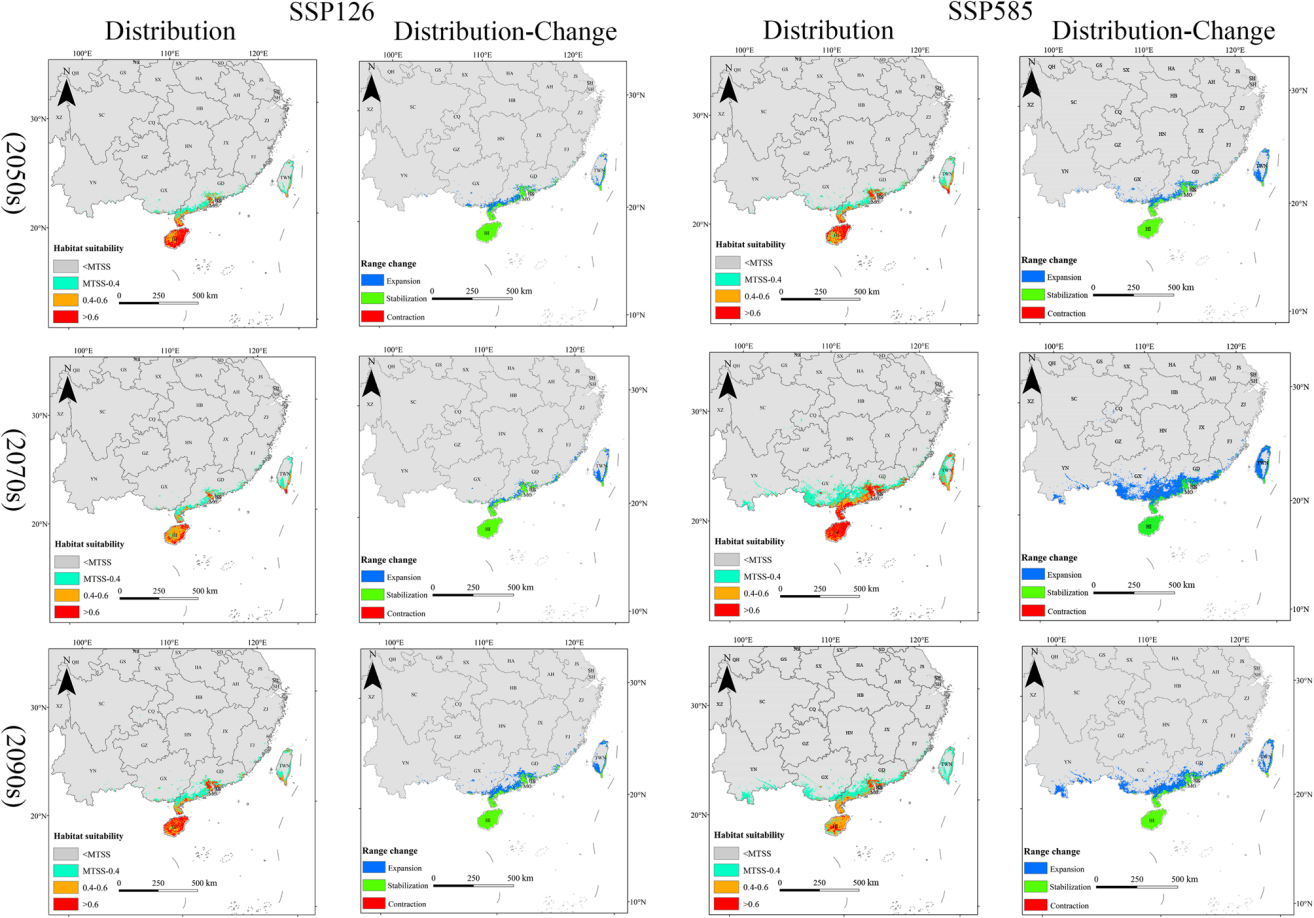
Projected future distributions of periods (2050s, 2070s, 2090s) for *O. arenosella* and the changes in areas between future and current distributions under two scenarios (SSP126 and SSP585).

In the context of climate change, the potential suitable habitats for *O. arenosella* in China are primarily located throughout Hainan Island and Taiwan, coastal regions of Guangdong and Guangxi, with sporadic distributions in Yunnan and Fujian (Fig. 6). There is an overall increase in the potential suitable habitat area for *O. arenosella* in future climate scenarios. This is mainly due to a reduction in the non-suitable areas and an increase in the high-, medium-, and low-suitability areas. Under the SSP126 (2090s) scenario, the areas with high, medium, and low suitability all increased. Similarly, under the SSP585 scenario, there is an overall increase in the potential suitable habitat area, characterized by increases in the high-, medium-, and low-suitability areas. Overall, the total suitable habitat area for *O. arenosella* is increasing (Table 2).

### Dynamic changes of the suitable area

Compared to the current, the overall suitable area for *E. torus Evans* remains relatively stable with minor variations within the range (Fig. 4). In the SSP126 scenario, compared to the current, the changes in suitable habitats for *E. torus Evans* in the 2050s and 2070s exhibit relative stability, with an equilibrium between expansion and contraction areas. From 2081 to 2100, there is a larger expansion area, primarily concentrated in the southeastern part of Yunnan Province, southern Guizhou, the entire Guangxi Province, Guangdong Province, Fujian Province, and some regions in Chongqing and Hubei. Under the SSP585 scenario, significant contractions could occur in 2090s, primarily occurring in the northwestern part of Yunnan Province, southeastern Guizhou Province, Guangxi Province, Guangdong Province, and Fujian Province. Overall, under both SSP126 and SSP585, the range of suitable areas for *E. torus Evans* does not change significantly.

Compared to the present, the overall suitable area for *R. ferrugineus* decreases, with some variations in its range, under climate change scenarios, and in these scenarios it is mainly concentrated in southeastern Tibet, Guangdong, Guangxi, Yunnan, Hunan, and Jiangxi (Fig. 5). Under SSP 126, most of the suitable areas remain stable in 2050s, with reductions mainly in the coastal areas of Jiangsu, Shanghai, Zhejiang, Yunnan, Guangxi, and eastern and southern Sichuan. Expansions are predicted in southeastern Tibet, southern Hubei, Chongqing, Jiangxi, Fujian, and Taiwan. In 2070s the suitable areas significantly contract, mainly in Zhejiang, Jiangxi, Fujian, Guizhou, Yunnan, Guangxi, Chongqing, and Sichuan, with minor expansions in southeastern Tibet, Hubei, and Zhejiang. Under SSP585, the suitable areas for *R. ferrugineus* contract considerably in 2050s, 2070s, and 2090s, primarily in Yunnan, Guangxi, Guangdong, Guizhou, Sichuan, Hunan, Zhejiang, Shanghai, and Jiangsu. Minimal expansion of suitable areas is predicted.

Compared to the present, the overall suitable area for the *O. arenosella* will significantly expand. Under climate change scenarios, the potential suitable areas for *O. arenosella* in China are mainly concentrated in the coastal areas of Guangdong, Guangxi, and Taiwan (Fig. 6). Under the SSP126 and SSP585 scenarios, the expansion of suitable areas mainly occurs along the coastal regions of Guangxi, Guangdong, and Taiwan. There are also some expansions in the southern part of Yunnan and the southeastern coastal areas of Fujian. Overall, there is a trend of northward expansion for *O. arenosella*.

### The Centroids Migration of the three invasive insects

The quantitative description of the centroid movement in potential suitable areas under climate change scenarios provides insights into habitat shifts for the invasive insect species. The current centroid of suitable habitats for *E. torus Evans* is located in the northeastern part of Guangxi Province (111.032664°E, 24.381571°N) (Fig. 2a). Under future climate scenarios, the centroid experiences comparatively smaller shifts, all within Guangxi Province. Overall, the centroid shifts toward the southwest direction under the SSP585 scenario, while it moves toward the south direction under the ssp126 scenario. Under SSP126, the centroid will move to the southwest (110.811127°E, 24.323439°N) during 2050s, southeast (111.193172°E, 24.272996°N) during 2070s, and northwest (111.151359°E, 24.322719°N) during 2090s. Under SSP 585, the centroid will move to the northeast (111.393446°E, 24.539894°N) during 2050s, southwest (111.151826°E, 24.194223°N) during 2070s, and continue southwest (110.891164°E, 24.129056°N) during 2090s.

The current centroid of suitable habitats for *R. ferrugineus* is located in the northern part of Guangxi Province (110.225126°E, 25.447637°N) (Fig. 2b). Under future climate scenarios, the centroid experiences relatively small shifts that are all within Guangxi Province. Overall, the centroid shifts toward the southwest direction under the SSP585 scenario and moves toward the east direction under the SSP126 scenario. Under SSP126, the centroid will move to the southwest (110.069562°E, 25.357406°N) during 2050s, southeast (110.14699°E, 25.184387°N) during 2070s, and northeast (110.389835°E, 25.302924°N) during 2090s. Under SSP585, the centroid will move to the southwest (109.903544°E, 25.083778°N) during 2050s, continue southwest (109.30784°E, 24.947314°N) during 2070s, and southeast (109.396298°E, 24.828778°N) during 2090s.

The centroid of suitable habitats for *O. arenosella* experiences simpler shifts, and is currently located in the northeastern maritime area of Hainan Province (111.133218°E, 20.40704°N) (Fig. 2c). Overall, the centroid shifts toward the northeast direction under both the SSP 585 and SSP 126 scenarios. Under SSP 126, the centroid will move to the northeast (111.985993°E, 21.151916°N) during 2050s, continue northeast (112.716081°E, 21.182953°N) during 2070s, and northwest (112.337654°E, 21.41479°N) during 2090s. Under SSP585, the centroid will move northeast (112.569632°E, 21.43052°N) during 2050s, northwest (111.679366°E, 22.171874°N) during 2070s, and southwest (111.352594°E, 21.798452°N) during 2090s.

### Key environmental variables

Internal jackknife tests of the MaxEnt model reveal the importance of several factors (Table 3). The precipitation of the coldest quarter (bio19, 31.5%), PD (31.4%), annual mean temperature range (bio7, 27.1%), and HII (4.7%) collectively contribute to 94.7% of the *E. torus Evans* distribution model. Additionally, PD accounts for 5.3% of the overall contribution. For *R. ferrugineus*, mean temperature of the driest quarter (bio9, 45.1%), precipitation of the driest month (bio14, 16.6%), temperature seasonality (bio4, 9.5%), and HII (8.2%) account for 79.4% of the distribution model. For *O. arenosella*, the minimum temperature of the warmest month (bio 6, 88.2%), PD (4.3%), precipitation of the warmest quarter (bio18, 2.5%), and mean diurnal range (bio2, 1.3%) account for 96.3% of the distribution model.

**Table 3:** Percentage contributions of the bioclimatic variables included in the Maxent models for *E. torus Evans*, *R. ferrugineus* and *O. arenosella* in China.

Species	Ranking of the importance of variables	First	Second	Third	Fourth	Fifth	Sixth	Seventh	Eighth	Ninth	Tenth	Eleventh	Twelfth	Thirteenth	Fourteenth	Fifteenth
*Erionota torus Evans*	Variable	bio19	PD	bio7	HII	DEM	NDVI	bio12	Slope	bio3	bio13	bio2	bio1	LC	LUCC	/
	Percent contribution	31.5	31.4	27.1	4.7	1.2	0.8	0.8	0.6	0.5	0.4	0.4	0.4	0.2	0.1	/
*Rhynchophorus ferrugineus*	Variable	bio9	bio14	bio4	HII	PD	bio2	bio15	DEM	bio8	LUCC	bio16	NDVI	LC	bio10	Slope
	Percent contribution	45.1	16.6	9.5	8.2	5.3	4.8	1.8	1.5	1.5	1.4	1.2	0.9	0.9	0.6	0.6
*Opisina arenosella*	Variable	bio6	PD	bio18	bio2	DEM	Aspect	NDVI	bio8	LC	LUCC	bio19	HII	bio15	/	/
	Percent contribution	88.2	4.3	2.5	1.3	0.9	0.5	0.4	0.4	0.4	0.4	0.4	0.2	0.1	/	/

*bio1* Annual mean temperature, *bio2* Mean diurnal range, *bio3* Isothermality, *bio4* Temperature seasonality, *bio5* Maximum temperature of warmest month, *bio6* Minimum temperature of warmest month, *bio7* Annual mean temperature range, *bio8* Mean temperature of wettest quarter, *bio9* Mean temperature of driest quarter, *bio10* Mean temperature of warmest quarter, *bio11* Mean temperature of coldest quarter, *bio12* Annual precipitation, *bio13* Precipitation of wettest month, *bio14* Precipitation of driest month, *bio15* Precipitation seasonality, *bio16* Precipitation of wettest quarter, *bio17* Precipitation of driest quarter, *bio18* Precipitation of warmest quarter, *bio19* Precipitation of coldest quarter, *HII* Human influence index, *NDVI* Normalized difference vegetation index, *LC* Land cover data, *DEM* digital elevation model data, *LUCC* the Land use and cover change, *PD* Population density.

## Discussion

### Model accuracy and uncertainty

To mitigate the impact of sampling bias on model predictions^45,48^, we conducted a screening process for the distribution points of the three invasive insects. To avoid multicollinearity issues among environmental variables^57^, environmental variables with an absolute correlation value less than 0.80 were selected using the stepwise method. When using Maxent to predict the potential distribution of *E. torus Evans*, *O. arenosella*, and *R. ferrugineus*, the value of the evaluation methods indicated the overall high performance of our model, which was adequate for predicting the suitable habitats of them (Table 4). Under current and future climate conditions, all model groups exhibited an AUC value exceeding 0.92, and the ROC curves extended toward the upper left corner, indicating excellent model prediction performance^51^. The AUC values were used to evaluate the accuracy of the model. The closer the AUC value is to 1, the higher the accuracy of the model prediction, and the closer the results predicted by the model are to the real state of the species distribution^51^. The validation results of the final model predictions consistently showed a close match between the test omission rate and the theoretical omission rate for *E. torus Evans, R. ferrugineus*, and *O. arenosella*. This indicated the absence of spatial autocorrelation between the selected climate variables and species distribution points. The contribution of the top four environmental variables used in the model exceeded 79.4% (94.7% for *E. torus Evans*; 79.4% for *R. ferrugineus*; 96.3% for *O. arenosella*), indicating appropriate selection of environmental variables^81^. Therefore, the predictive results of this study are accurate and reliable, enabling analysis of the potential distribution of *E. torus Evans*, *R. ferrugineus*, and *O. arenosella* in their suitable habitats in China.

**Table 4:** The AUC values of three invasive insects under the future climatic scenarios.

Climate change scenario	Year	*Rhynchophorus ferrugineus*	*Erionota torus Evans*	*Opisina arenosella* walker
	Current	0.97	0.987	0.994
SSP126	2050s	0.975	0.991	0.995
	2070s	0.973	0.989	0.992
	2090s	0.974	0.924	0.995
SSP585	2050s	0.973	0.989	0.994
	2070s	0.973	0.987	0.995
	2090s	0.973	0.988	0.994

**Figure 7:**
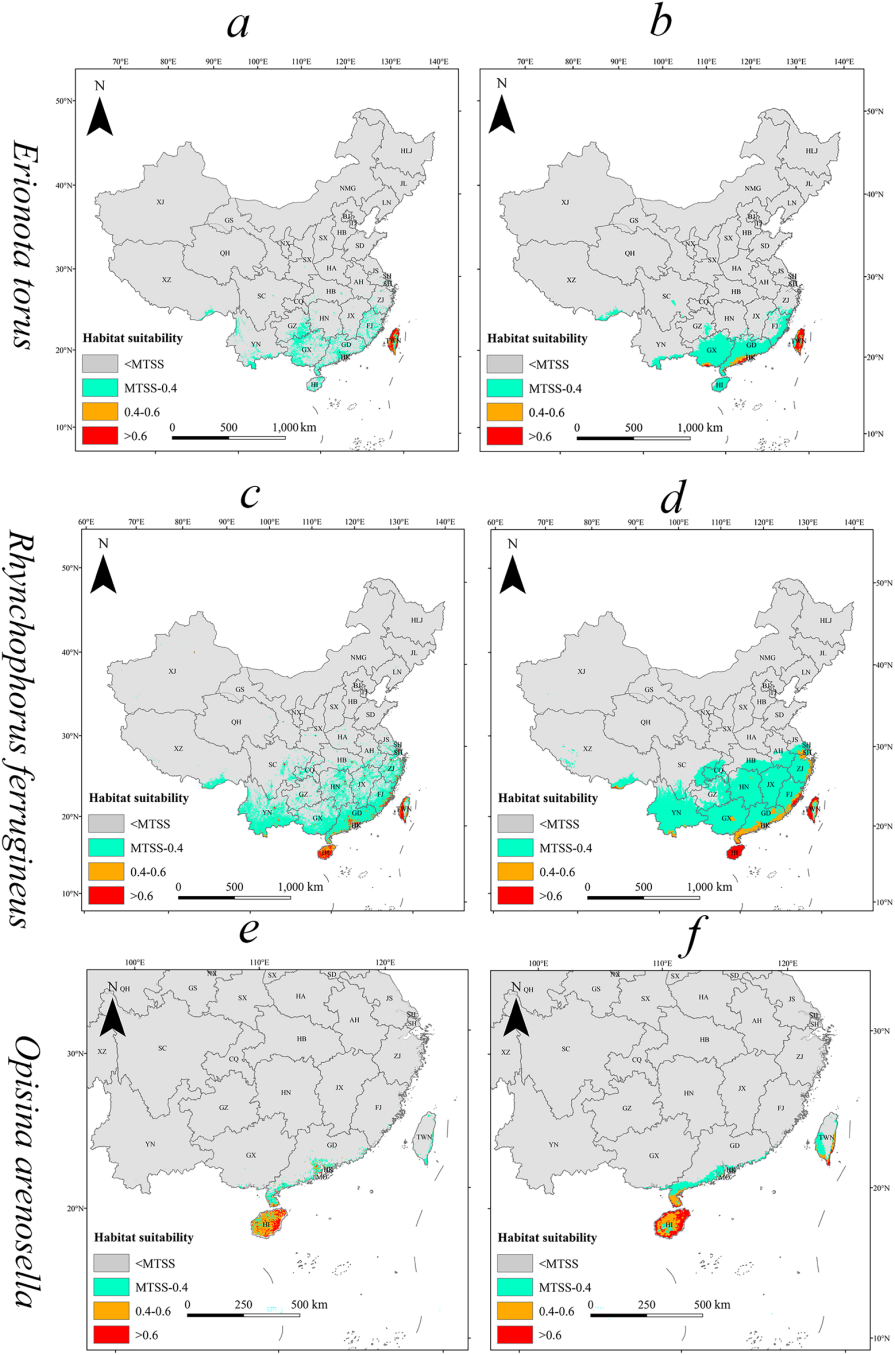
The distribution of potential suitable areas based all types of environmental variables including climate factors (Left) and the distribution of potential suitable areas based solely on climatic factors (Right) under the current climatic conditions for (**a**) *E. torus Evans*, (**b**) *R. ferrugineus*, (**c**) *O. arenosella*.

Compared to predictions based solely on climatic factors, the predictions incorporating other variables resulted in a reduction and discontinuity in the range of suitable habitats (Fig. 7). A study on the distribution range of *Apis dorsata* revealed that the inclusion of additional environmental variables (such as terrain factors, human disturbance factors, and habitat factors) in the simulation results led to a notable decrease and fragmentation of its potential suitable habitats^45^. Our findings also suggest that these factors may have a significant impact on the distribution of the three invasive insects and resulted in the decrease and fragmentation of their potential suitable habitats. Many factors affect species distribution, in addition to the factors mentioned above, we should consider incorporating the biological characteristics of the species into the model construction in the future. Biological characteristics are intrinsic factors that influence the distribution of invasive species^55^.

### Characteristics of the current potential distributions

Under the current climatic conditions, the potential suitable habitats of *E. torus Evans* are concentrated in the southwestern region of China and the coastal areas of the southeast, including Yunnan, Guizhou, Guangdong, Guangxi, Fujian, Hainan, and Taiwan. These regions have a low-latitude monsoon climate characterized by abundant summer rainfall and relatively high temperatures^65,66^. Temperature and humidity ranges are crucial factors influencing its distribution^67^. This is consistent with previous research, indicating a strong agreement between the simulated potential geographic distribution of *E. torus Evans* and its actual range under the current climatic scenarios^67^. The main distribution areas of *R. ferrugineus* encompass a large portion of southern China, including Hainan, Guangxi, Guangdong, Hunan, Jiangxi, Fujian, Yunnan, Guizhou, Hubei, Anhui, Jiangsu, as well as most of the southeastern coastal regions and Taiwan. These areas provide highly favorable habitats, which cover the current distribution of the species. This finding aligns with previous research, suggesting that the high adaptability of *R. ferrugineus* may explain its wide distribution^22^. *O. arenosella* is primarily distributed in southern coastal areas, including Hainan, Guangxi, Guangdong, and Taiwan. The predicted distribution range differs from previous reports^68^, possibly due to our consideration of additional environmental variables, such as the influence of human activities. Previous studies were based on global distribution points of *O. arenosella* for prediction, while our research is based on existing distribution points in China. This resulted in a prediction that better aligns with the actual distribution of this species in China. These factors play a significant role in the distribution of *O. arenosella* and may contribute to the disparities in distribution predictions compared to previous studies^68^.

Among the three invasive insects, the distribution ranges of *E. torus Evans* and *R. ferrugineus* are relatively broader compared to *O. arenosella*. This may be because *E. torus Evans* invaded Taiwan before 1986 and was subsequently discovered in southern China^10^. *R. ferrugineus* was first detected in Guangdong Province in 1997 and has become widely distributed in various southern regions of China over the past 30 years due to palm plant transportation^69^. *E. torus Evans* and *R. ferrugineus* may have become better adapted to the invaded environment due to their longer invasion history^33,67,69^. However, *O. arenosella* was only recently discovered in Wanning City (2013). As a result, its invasion history is relatively short, and there has not been a large-scale invasion^32^.

### Dynamic distribution of three invasive insects

Under different climate scenarios, there was no significant change in the suitable habitat area for *E. torus Evans*, while the suitable habitat area for *R. ferrugineus* decreased, and a significant expansion was observed for *O. arenosella*. Although the direction of changes in suitable habitats for *E. torus Evans* under different future climate scenarios has not shifted in the same direction and there is a scattered northward expansion trend in the central region of China, the predicted results consistently indicate that regions such as Guizhou, Guangxi, Guangdong, Fujian, Hainan, and Taiwan remain high-suitability areas for this species. The frequent economic and trade exchanges between most cities in these regions and other regions are conducive to the spread of *E. torus Evans*. Therefore, in the future it is crucial to strengthen risk monitoring and control measures in these areas. Overall, the general suitable habitat of *R. ferrugineus* shows a reduction trend, consistent with previous research^22^. A similar phenomena has also been observed in *S. geminata*^43^. This could be attributed to the combined effects of future temperature and precipitation factors. The suitable habitat area for *O. arenosella* undergoes expansion but remains limited to the southern coastal areas and Taiwan. This may be related to their climatic requirements and dietary preferences^31^. Global climate warming and rising temperatures promote reproduction and expansion of the *O. arenosella*'s overwintering range^38,39^. As *O. arenosella* primarily inhabits leafy forests in coastal areas^32^, we conclude that the potential suitable habitats for this species will continue to expand. Therefore, it is necessary to strengthen detection and control of *O. arenosella* in these regions.

### Key drivers of the invasive alien insects

We found that among the top four environmental variables that contributed to the distribution of these three invasive species, temperature contributed the highest, followed by precipitation, and finally HII and PD. In other words, temperature, precipitation, and human disturbances (PD, HII) are key factors influencing the distribution of the three invasive species. The most important environmental factors are temperature and precipitation. PD and the HII are more important variables. Terrain and habitat factors are less influential. The findings is supported by research on other invasive insect species^70,71^.

Insects are ectothermic animals that are highly sensitive to temperature changes. Temperature can directly affect their growth, development, reproduction and survival^72^. The development time of *E. torus Evans* is related to temperature^67^. The ambient temperature needs to reach above 4.10 ± 0.56 °C for *R. ferrugineus* to complete the egg stage. Similarly, the ambient accumulated temperature can only meet the generation or life history requirements when it is higher than 1067.7 days °C^23^. The larval development time and adult lifespan of *O. arenosella* are closely related^33^, Low temperatures can slow population growth and extend egg hatching time^73,74^, while summer heat favors the growth of *O. arenosella*^75^. Moreover, higher temperatures can improve insect overwintering survival rate, unsuitable habitats for insects are transformed into habitats suitable for their survival^76^. This may explain why the suitable habitats and centroid of *O. arenosella* shifted to high-latitude areas under future climate scenarios*.* Previous studies indicate that the overall distribution range of *R. ferrugineus* has decreased^22^. Our research also found that the suitable habitat area of *R. ferrugineus* showed an overall contraction, but there were expansions of suitable habitats in certain areas, which could be attributed to reduced cold stress in these regions as temperatures rise. The increase in extreme weather events, such as floods and droughts, as a result of climate warming, may lead to a decrease in the number and potential distribution of invasive species^38^, which also explains why the geographical distribution of the three invasive insect species will shrink under certain future climate scenarios.

Precipitation can affect insect development time, pathogen transmission, and nest security, while precipitation itself can directly affect the lives of small insects^77^. Precipitation also changes the relative humidity of the air, which affects host plants and indirectly affects insects^77^. For example, from August to October in Guangzhou, frequent typhoons and heavy rains reduced the density of insect populations for *E. torus Evans* in the fields. Among the environmental variables influencing *E. torus Evans*, Precipitation of the coldest quarter (bio19) ranks first, which is further evidence that precipitation has a great influence on the geographical distribution of this insect.

Human activities have also a significant impact on the changes in suitable habitats for *E. torus Evans*, *O. arenosella*, and *R. ferrugineus*. This is consistent with previous descriptions of how human activities can influence their expansion^78,79^. Under favorable climate conditions, human activities may accelerate insect spread in invaded areas. Since these three invasive insects primarily rely on palm plants as their main food source^67,68,80^, modern intensive palm cultivation provides an ideal ecological habitat for pests and increases the risk of insect transmission. Other human disturbances, such as trade activities and urban greening, also contribute to the distributions of these three invasive insects. In other words, human activities break down the geographical limits of species, resulting in increased biological invasions. Moreover, human activities cause climate warming, affecting temperature and precipitation, which in turn affects the geographical distribution of invasive species.

### Measures for the control and management

At present, globalized economy and climate warming have promoted the global successful invasion of invasive alien species^81^. Managing the global invasive species in the face of climate change has become extremely difficult, which is also confirmed by our findings that the interaction between climate and human activities determines the geographical distribution of these three invasive insect species. *E. torus Evans*, *R. ferrugineus* and *O. arenosella* can disperse over long distances through inter-city trade of crops, and Ornamental plants such as banana, palm trees, and coconut palms. Our results showed that human factor has great influence on the distribution of invasive insects, thus in view of the areas with high Population density and HII, the government departments should regulate human activities to reduce the migration of invasive insects. Government departments should also strengthen publicity, management and epidemic prevention with the adjustment of agricultural practices or trade regulations. The area with high potential for the three invasive insects to invade is the most suitable area for their reproduction. Different prevention and control strategies should be adopted in different adaptive areas. For *E.torus Evans*, prevention and control should be strengthened in Taiwan, Guangxi, Guangdong, and Fujian; for *O.arenosella* prevention and control should be strengthened along the coasts of Hainan and Guangxi; for *R. ferrugineus*, prevention and control should be strengthened in southern Yunnan, Hainan Island, Taiwan, and the southeastern coastal regions. Furthermore, the area where the three invasive species have erupted in large numbers, mechanical, chemical, and biological control measures should be implemented immediately to destroy it. Finally, applying intensive control at early stages generally increased the effectiveness of control^81^. Thus, it is necessary to organize relevant units and personnel to carry out a large-scale investigation in areas with high invasion potential and expected to see significant habitat expansions to determine the quantity and harm status of the species, so that to provide scientific basis for taking corresponding prevention and control measures. The measures mentioned above will enrich the international body of knowledge on invasive species management.

### Existing deficiencies and future development directions

Although our study takes into account as many factors as possible that affect the distribution of *E. torus Evans*, *O. arenosella*, and *R. ferrugineus*, there are still many shortcomings. Firstly, species distribution is subject to interactions between biological and abiotic factors^82^. Our study mainly considered the influence of climate, PD, HII, LC, LUCC, NDVI, slope, aspect, and DEM on the distribution of the three invasive species. However, the interaction between organisms, transportation construction, trade activities, water conservancy projects, and so on play an important role in the distribution of invasive species^51^. Therefore, the interaction between invasive species and native biota, the socio-economic implications of invasive species management and so on should be considered into the model, and more accurate prediction results may be obtained. Secondly, screening of life-history attributes in the invasion phases of spread will further improve the accuracy of prediction. This study used long-term data with 20-year intervals that might not capture rapid evolutionary changes or species' behavioral adaptations to new environments. Thirdly, although the distribution sites of these three invasive species have been obtained as much as possible, the actual distribution points of the species cannot be fully collected. Future environmental variables used, such as LC, PD, LUCC, NDVI, may differ from what is true in the future. It is impossible to accurately predict the changes of HII, LC, PD, LUCC and NDVI, so these data were invariant in the future in the present study, (2050s, 2070s, 2090s).

## Conclusions

We employed the MaxEnt model to predict the potential distribution dynamics centroid transfer of three invasive insects, *Erionota torus Evans*, *Rhynchophorus ferrugineus*, and *Opisina arenosella* walker under current and future climate scenarios, while investigating the key environmental factors influencing their distribution. All model groups exhibited an AUC value exceeding 0.92, indicating excellent model prediction performance. The suitable habitat area for *E. torus Evans* remained relatively stable, while *R. ferrugineus* had a decrease in suitable habitat area in both climate models. *O. arenosella* exhibited a significant expansion in its suitable habitat area. Due to their longer history in China following invasion, *E. torus Evans* and *R. ferrugineus* may have evolved significant adaptation to the local environment. The current centroid of suitable habitats for *R. ferrugineus* and *E. torus Evans* is located in Guangxi Province and wholely shift toward the south direction under future climate. The suitable habitats and centroid of *O. arenosella* shifted to high-latitude areas under future climate scenarios. It is necessary for relevant government departments to carry out a large-scale investigation in areas with high invasion potential and expected to see significant habitat expansions to determine the quantity and harm status of the species, so that to provide scientific basis for the development of early warning systems and taking corresponding prevention and control measures.

The research findings also indicate that temperature, precipitation, population density, and the Human influence index are the primary driving factors influencing the potential distribution o+f these invasive insects. The most important environmental factors are temperature and precipitation. Population density and Human influence index are more important variables. Human activities, including agricultural practices, trade activities, and urban greening exert substantial influences on the changes in suitable habitats for these species. In order to reduce the spread of these invasive insects, policies should be developed to regulate agricultural practices and invasive species trade between cities by government departments. This study relies on long-term climatic and bioclimatic data and may not account for short-term ecological changes or species' behavioral adaptations. The factors influencing the distribution of these invasive species considered in our study are limited. So the interaction between invasive species and native biota, the socio-economic implications of invasive species management and so on should be considered when studying the distribution of these species in the future.

This work is necessary and timely in identifying newly areas at high risk of expansion of the three invasive insects in the future, then suggesting strategic control measures to prevent their spread, and finally providing scientific evidence for the early prevention and rapid response to the three invasive insects. We hope that the management authorities will take active measures to strengthen the prevention and control management of these three invasive species, because this will not only contribute to the conservation of biodiversity and agricultural production, but also contribute to achieve the United Nations' Sustainable Development Goals.

## Data Availability

The datasets generated and/or analysed during the current study are not publicly available due (our experimental team's policy) but are available from the corresponding author on reasonable request.
